# The Fate of Bacteria in Human Digestive Fluids: A New Perspective Into the Pathogenesis of *Vibrio parahaemolyticus*

**DOI:** 10.3389/fmicb.2019.01614

**Published:** 2019-07-16

**Authors:** Siqi Wang, Zhaohuan Zhang, Pradeep K. Malakar, Yingjie Pan, Yong Zhao

**Affiliations:** ^1^College of Food Science and Technology, Shanghai Ocean University, Shanghai, China; ^2^Laboratory of Quality and Safety Risk Assessment for Aquatic Products on Storage and Preservation, Ministry of Agriculture and Rural Affairs of the People’s Republic of China, Shanghai, China; ^3^Shanghai Engineering Research Center of Aquatic-Product Processing and Preservation, Shanghai, China

**Keywords:** *Vibrio parahaemolyticus*, digestion fluids, survival, pathogenesis, escape mechanism

## Abstract

*Vibrio parahaemolyticus* causes the most seafood-attributed gastroenteritis outbreaks worldwide and studies on its pathogenesis during passage through the human digestive fluids are limited. An *in vitro* continuous model system mimicking passage through saliva, gastric and intestinal fluid was used to study the survival, morphology and virulence-related gene expression of a total of sixty pathogenic, and non-pathogenic *V. parahaemolyticus* strains. The changes to these three characteristics for the sixty *V. parahaemolyticus* strains were minimal on passage through the saliva fluid. No *V. parahaemolyticus* strains survived passage through gastric fluid with low pH values (2.0 and 3.0) and the cells, examined microscopically, were severely damaged. However, when the pH of gastric fluid increased to 4.0, the bacterial survival rate was 54.70 ± 1.11%, and the survival rate of pathogenic strains was higher when compared to non-pathogenic strains. Even though the bactericidal effect of intestinal fluid was lower than gastric fluid, virulence-related gene expression was enhanced in the intestinal fluid. Seafood matrices can significantly raise the pH level of gastric fluid and thus aid the survival of *V. parahaemolyticus* through passage from human gastric acid and progression of pathogenesis in the intestinal fluid. We confirmed these phenomena in the *in vitro* continuous digestion model.

## Introduction

*Vibrio parahemolyticus* is the most common foodborne pathogen found in the marine and estuarine environment (Robert et al., 1969). Consumption of raw or undercooked seafood contaminated with high numbers of this bacterium causes acute gastroenteritis and serious septicemia (McLaughlin et al., 2005). The two major toxins of *V. parahaemolyticus* are heat resistant direct hemolysin (TDH) and heat resistant hemolysin related hemolysin (TRH), which are encoded by *tdh* and *trh* genes, respectively (Raimondi et al., 2000; Ceccarelli et al., 2013). TRH and TDH have high sequence homology (68%) where these toxins show beta-hemolytic activity on Wagatsuma agar and both show cytotoxicity ad enterotoxicity (Takeshi et al., 1990; Ohnishi et al., 2011). A type III secretion system (T3SS), which can utilize a needle-like apparatus to translocate effectors into the host cells, also contributes to the virulence of *V. parahaemolyticus* (Ham and Orth, 2012; Zhang and Orth, 2013). T3SS2 is associated with cytotoxicity in intestinal cells and enterotoxicity in infected animal models (Ham and Orth, 2012). However, there is limited information of the pathogenesis of *V. parahaemolyticus* through passage in the human digestive tract.

Takaaki used Caco-2 cells to reveal the infection pathway of *V. parahaemolyticus* (Shimohata et al., 2011) and a rabbit model was used to reveal how *V. parahaemolyticus* causes intestinal epithelial surface damage and progression of pathogenesis (Ritchie et al., 2012). However pathogenesis is dependent on *V. parahaemolyticus*, which in spite of being acid sensitive, survives the low pH in the gastric fluid. Survival and escape from the gastric fluid can be studied using an *in vitro* simulation model of digestive processes. This model system simulates pH changes, enzymes and gastric emptying, gastric peristalsis, secretion of digestive fluids, and various other dynamic processes (Molly et al., 1993; Havenaar and Minekus, 1996; Barroso et al., 2015).

This study aims to determine the survival rates, morphological changes, survival variability and related virulence gene expressions of *V. parahaemolyticus* in simulated digestive fluids (Saliva, Gastric fluid, and Intestinal fluid). This information will improve the understanding of the pathogenesis of *V. parahaemolyticus* in human digestive tract and provide information for design of additional control measures to reduce the human disease burden.

## Materials and Methods

### *Vibrio parahaemolyticus* Strains and Inoculum Preparation

The strain information is shown in Supplementary Table 1. The sixty strains of *V. parahaemolyticus* used in this study were collected from the American type culture collection (ATCC, 2 strains), from patients admitted to Shanghai hospital (28 strains), and from aquatic products sold from retail outlets in Shanghai (30 strains). Strains were stored at -80°C in glycerol test tubes with Tryptic Soy Broth (TSB, Land Bridge Technology, Beijing, China) and 50% (v/v) glycerol. Strains were steaked onto thiosulfate citrate bile salts sucrose (TCBS; Beijing Land Bridge Technology Company Ltd., Beijing, China) agar culture medium and incubated at 37°C for 18 h. A single colony from TCBS plate was transferred into 9 mL TSB containing 3% NaCl, and then cultured overnight at 37°C and 220 rpm. The overnight culture was then diluted with 0.1% peptone water (PW; Beijing Land Bridge Technology Company Ltd., Beijing, China) to an optical density value for 1.2 ± 0.02 at 600 nm (OD_600_). A further 100 fold dilution with 0.1% PW diluted was needed to obtain the appropriate number of bacteria for the digestion experiments. All the experiments were carried out in a Class 2 biological safety cabinet, where appropriate.

### Preparation of Simulated Human Digestion Fluids and Detailed Digestion Protocol

**Figure 1:**
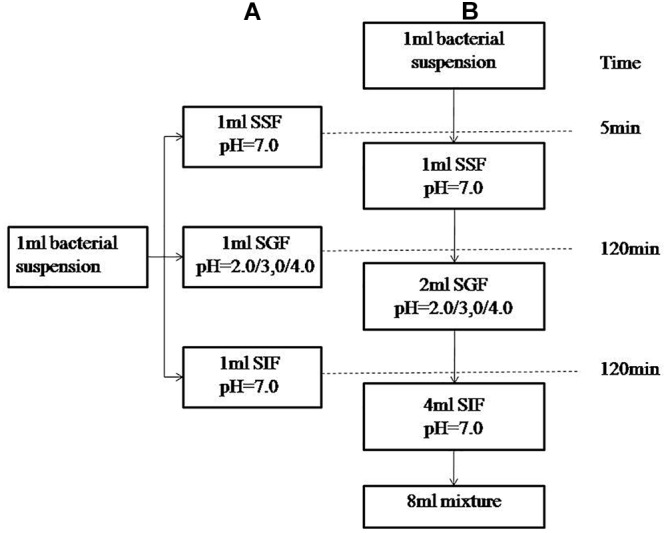
Schematic representation of the experimental designs. **(A)** describes experiment in which the bacterial suspensions were treated with simulated salvia fluid (SSF), simulated gastric fluid (SGF), and simulated intestinal fluid (SIF). **(B)** describes a three-step *in vitro* continuous digestion model. All the experiments were conducted at 37°C and at a shaking at the speed of 110 rpm.

The simulated human digestion fluids included saliva (SSF), gastric fluid (SGF), and intestinal fluid (SIF). Formulation of simulated digestion fluids as described by Minekus (Minekus et al., 2014) is shown in Table 1. 1 M HCl and 1 M NaOH were used to adjust the pH (SSF at 7.0, SGF at 2.0/3.0/4.0, SIF at 7.0) and all of the fluids were pre-warmed at 37°C before use. Figure 1A shows the specific steps of digestive fluids treatment for *V. parahaemolyticus* and Figure 1B shows a detailed protocol of a simulated continuous human digestion model. Plate counting method was used to enumerate bacteria surviving the digestive fluids treatment. Each sample was plated in duplicate on TCBS after appropriate 10-fold serial dilution by 0.1% PW and incubated at 37°C for 24 h for colony counting.

**Table 1:** Formulation of simulated human digestion fluids.

Constituent conc.	SSF	SGF	SIF
KCl^1^	15.1 mmol L^-1^	6.9 mmol L^-1^	6.8 mmol L^-1^
KH_2_PO_4_^1^	3.7 mmol L^-1^	0.9 mmol L^-1^	0.8 mmol L^-1^
NaHCO_3_^1^	6.8 mmol L^-1^	12.5 mmol L^-1^	42.5 mmol L^-1^
NaCl^1^	–	11.8 mmol L^-1^	9.6 mmol L^-1^
MgCl_2_(H_2_O)_6_^1^	0.5 mmol L^-1^	0.4 mmol L^-1^	1.1 mmol L^-1^
(NH_4_)_2_CO_3_^1^	0.06 mmol L^-1^	0.5 mmol L^-1^	–
Salivary α-amylase^2^	150 U ml^-1^	–	–
pepsin^3^	–	4000 U ml^-1^	–
trypsin^4^	–	–	200 U ml^-1^
bile^5^	–	–	0.02 mmol L^-1^
CaCl_2_(H_2_0)_2_	1.5 mmol L^-1^	0.15 mmol L^-1^	0.6 mmol L^-1^

*The standard formulation was supplemented with CaCl_*2*_(H_*2*_0)_*2*_ to prevent precipitation. 1, the constituents were all sourced from Land Bridge Technology, Beijing, China; 2, α-amylase from human saliva, Type IX-A, 1000–3000 units/mg protein, Sigma; 3, Pepsin from porcine gastric mucosa, ≥250 units/mg, Sigma; 4, trypsin from bovine pancreas, ≥25000 units/g, Sigma; 5, Bile salts, Sigma*.

### Preparation and Inoculation of Aquatic Product Samples

Samples of fresh salmon, oysters and shrimps were purchased from a local supermarket in Shanghai, China, and stored at -20°C before treatment. After thawing, seafood samples were put into boiling water for 20 min to inactivate the native bacteria and then cooled to room temperature (25 ± 2°C) (Xie et al., 2012). A food processor (CombiMax K600, Braun, Germany) was used to reduce the particle size of the seafood samples as an adjunct to chewing. The speed of the chopping blade was set at 1500 rpm for 5 min to prepare the “chewed” salmon/shrimp/oysters (150 g sample and 20 ml sterile water) (Agrawal et al., 1997; Hermans et al., 2012; Lewis et al., 2012; Qing et al., 2013). Each chewed sample was inoculated with 1 ml of culture suspension (10^9^ CFU/ml) for 30 min to allow for bacterial attachment at room temperature. The final concentration of *V. parahaemolyticus* inoculated on the seafood samples was 7 log_10_ CFU/g on average and each sample was prepared in duplicate. The inoculated aquatic products were mixed with SGF (pH = 1.6) in a ratio of 1:1 and cultured in a shaker which was set to 110 rpm and 37°C for 2 h.

### Scanning Electron Microscopy (SEM) and Transmission Electron Microscopy (TEM)

In preparing a sample for SEM, 1 ml of bacteria suspension was fixed with 2.5% glutaraldehyde for 4 h at 4°C, and this sample was then dehydrated in a solution containing ascending concentrations of ethanol (30, 50, 70, 90, and 100% twice for 10 min each). A FEI Nova 450 scanning electron microscope was then used to observe the bacteria after the samples were gold plated. In prepping a sample for TEM, a 1% osmium acid solution was used to fix the sample for 2 h and dehydrated as described above. The dehydrated sample was then treated with a mixture of entrapped agents and acetone (V/V = 1/1) for 1 h and a Leica UC6 ultramicrotome was used to obtain ultrathin slices. These ultrathin slices were treated further with uranyl acetate solution saturated with 50% alcohol and lead citrate solution, respectively for 5 min before observation under a JEM2100 transmission electron microscope.

### RNA Extraction and RT-PCR

RNA was extracted using a RNA Extraction Kit (Shanghai general Biotechnology Co., Ltd.) in accordance with the manufacturer’s instructions. The concentration of RNA was determined using absorbance at 260 nm (ND-1000 spectrophotometer, NanoDrop Technologies, Wilmington, DE, United States) and 200 ng total RNA was used for reverse transcription by RT kit and gDNA Eraser (Takar, Dalian, China) also in accordance with the manufacturer’s instructions.

Table 2 lists the gene function and the primers used for qPCR. The final volume of each qPCR reaction mixture was 20 μl and was made up with 0.4 μl ROX Π, 10 μl TaqMan Gene Expression Master Mix, 6 μl ddH_2_O, 2 μl of template cDNA, and 0.8 μM of forward and reverse PCR primers. Deionized water was used as a negative control in each operation. Duplicate reactions for the gene of interest and the 16S rRNA gene were run in the Applied Biosystems 7500 Fast Real-Time PCR System (Applied Biosystems, Carlsbad, CA, United States). Relative quantification was analyzed by the comparative CT method (ΔΔCT method) (Livak and Schmittgen, 2001).

**Table 2:** Gene function and primer sequences for the RT-qPCR assay.

Gene	Biological acitivity	Primer sequences (5′ – 3′)	References
*16S*	Control gene	F – GTTGGTGAGGTAAGGGCTCA	Wang et al., 2013
		R – GCTGATCATCCTCTCAGACCA	
*tdh*	Forms pores on cells	F – GTAAAGGTCTCTGACTTTTGGAC	Feng et al., 2016
		R – TGGAATAGAACCTTCATCTTCACC	
*trh*	Forms pores on cells	F – TTGGCTTCGATATTTTCAGTATCT	Feng et al., 2016
		R – CATAACAAACATATGCCCATTTCCG	
*vopZ(vpa1336)*	Enterotoxicity and clonization	F – AGAGGATGAGGATGAGGAGTGTG	GeneBank database
		R – TCGATTGCTGATACTGCAGCG	
*vopL(vpa1370)*	Induces stress fiber	F – GCCACTACCATCAGCGTCAACAAG	GeneBank database
		R – CATGTGTCGTCGGCTCGAACG	
*vopV(vpa1357)*	Facilitates enterotoxicity	F – TCGTCGTGAACCAGAGCCTGAG	GeneBank database
		R – CGTGTCCGCCTGATTCGTCTTATC	
*vtrA*	Activates the T3SS	F – TTGGAACCCACGAACATCTC	Li et al., 2016
		R – CAGTCACAAATTTTCCTGGCC	
*vtrB*	Activates the T3SS	F – ATTATCAGCTTAGGTGGGCG	Li et al., 2016
		R – ACTTTACCCCACACTTTGTCG	

### Statistical Analysis

All data are presented as the mean ± standard deviation (SD). Statistical analysis was performed using SPSS statistical package 19.0 (SPSS Inc., Chicago, United States). The level of statistical significance (*p* < 0.05) was tested by analysis of variance (ANOVA).

## Results

### Survival and Morphological Changes of *V. parahaemolyticus* in Simulated Digestion Fluids

**Figure 2:**
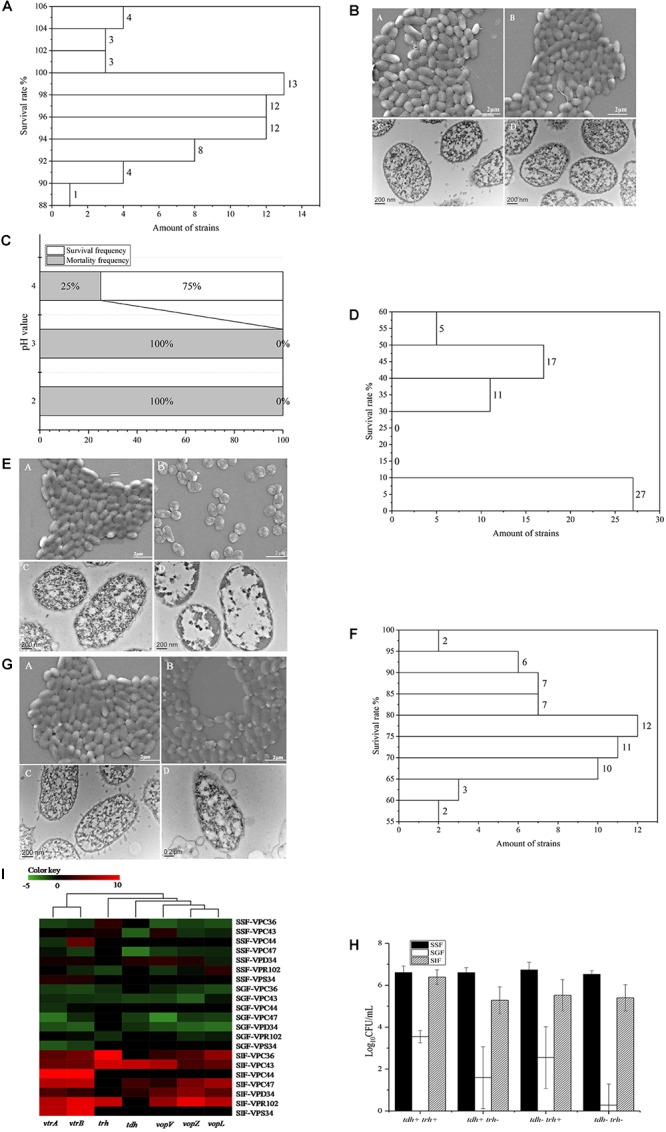
Survival of *V. parahaemolyticus* in simulated digestive fluids. **(A)** Distribution of *V. parahaemolyticus* viability [treatment (Log_10_CFU/mL)/control (Log_10_CFU/mL)] treated by SSF. **(B)** Representative photomicrographs of untreated *V. parahaemolyticus* by SEM **(A)** and TEM **(C)**, *V. parahaemolyticus* treated by SSF by SEM **(B)** and TEM **(D)**. **(C)** Mortality and survival frequency distribution of *V. parahaemolyticus* treated with SGF (pH = 2.0, 3.0, and 4.0). **(D)** Distribution of *V. parahaemolyticus* viability [treatment (Log_10_CFU/mL)/control (Log_10_CFU/mL)] treated by SGF (pH = 4.0). **(E)** Representative photomicrographs of untreated *V. parahaemolyticus* by SEM **(A)** and TEM **(C)**, *V. parahaemolyticus* treated by SGF by SEM **(B)** and TEM **(D)**. **(F)** Distribution of *V. parahaemolyticus* viability [treatment (Log_10_CFU/mL)/control(Log_10_CFU/mL)] treated by SIF. **(G)** Representative photomicrographs of untreated *V. parahaemolyticus* by SEM **(A)** and TEM **(C)**, *V. parahaemolyticus* treated by SIF by SEM **(B)** and TEM **(D)**. **(H)** Survival of *V. parahaemolyticus* from different genotypes in simulated digestive fluids, respectively. **(I)** Heatmap shows the relative expression of virulence-related genes in *V. parahaemolyticus* in simulated digestive fluids for 2 h, respectively. The color codes represent relative expressions, ranging from green (induced expression) to red (repressed expression).

The survival of *V. parahaemolyticus* in SSF, is shown in Supplementary Table 2. The viable cell counts between control and treatment groups were not significantly different (*P* > 0.05). The survival rate of the 60 strains of *V. parahaemolyticus* ranged from 105.41 ± 0.43% to 89.46 ± 0.44%. The frequency distribution of bacterial viability after simulated saliva treatment is shown in Figure 2A, of which 10 isolates were over 100% and only 1 was below 90%. Both SEM and TEM images (Figure 2B) showed that SSF treatment had no significant effect on the viability of *V. parahaemolyticus*.

Three different gastric fluids, SGF, with pH values of 2.0, 3.0, and 4.0 were chosen to study the survival of *V. parahaemolyticus*. The results of viable cell counts are shown in Supplementary Table 3 and in this fluid, the viable cell counts between control and treatment groups were significantly different (*P* < 0.01). In SGF at pH 4.0, the maximum survival rate was 54.70 ± 1.11%. None of the sixty *V. parahaemolyticus* isolates could survive in the simulated gastric fluid (SGF) of pH 2.0 and 3.0, while 75% of isolates were still viable at pH 4.0 (Figure 2C). The frequency distribution of bacterial viability after SGF (pH = 4.0) treatment is shown in Figure 2D. SGF also induced a drastic change in the cell morphology of *V. parahaemolyticus* (Figure 2E). The cell morphology, observed using SEM, changed from typical smooth rod-shaped *V. parahaemolyticus* to shrunken spherical-shaped bacteria. These spherical-shaped bacteria, observed under TEM, showed a cytoplasm that was aggregated and which no longer had obvious functional zones.

The survival of *V. parahaemolyticus* in SIF is shown in Supplementary Table 4. Analysis of this data indicated that viable cell counts between control and treatment groups were significantly different (*P* < 0.05). In the treatment group the survival rate among the 60 strains of *V. parahaemolyticus* ranged from 96.16 ± 0.19% to 56.78 ± 0.81% and the frequency distribution of this bacterial viability is shown in Figure 2F. Since none of the isolates were above 100% and 52 isolates were below 90%, the bactericidal effect of SIF on *V. parahaemolyticus* was greater than that of simulated saliva fluid. Although no significant difference was observed between the SIF treated *V. parahaemolyticus* and the control group under SEM, examination of TEM images showed changes in vesicular structure, thinner cell walls, and increased cytoplasmic division (Figure 2G).

The 60 *V. parahaemolyticus* isolates can be classified into four different genotypes according to the type of virulence gene in the DNA, *tdh-/trh-*, *tdh-/trh+*, *tdh+/trh-*, and *tdh+/trh+*. Survival of *V. parahaemolyticus* of different genotypes in the 3 simulated human digestion fluids is shown in Figure 2H. The survival of *tdh+/trh+* strains was significantly higher than that of the other three genotypes and the *tdh-/trh-* was the most intolerant in SGF.

### *Vibrio parahaemolyticus* Virulence-Related Gene Expression in Simulated Digestion Fluids

Seven strains were selected for virulence gene expression experiment These were the VPC36 (*tdh-/trh+*), VPC43 (*tdh+/trh+*), VPC44 (*tdh-/trh-*), VPC47 (*tdh+/trh-*), VPD34 (*tdh+/trh-*), VPR102 (*tdh-/trh+*), and VPS34 (*tdh-/trh-*) strains. Transcription of genes evaluated in this study is strongly influenced by the digestive fluids (Figure 2I). In simulated digestive fluids, virulence-related genes were significantly up-regulated only after SIF treatment, and most of them were downregulated in SSF and SGF. Gene clusters using the UNIFRAC framework is shown in the Figure 2I. The 7 genes can be divided into two main branches. The first branch consists of *vtrA* and *vtrB* genes coding for the transcriptional regulatory proteins, VtrA, and VtrB. The second branch consists of the genes encoding T3SS2 effectors proteins, *vopV, vopZ*, and *vopL* and the toxin genes *trh, tdh.* This second branch genes encode proteins which are related to cytotoxin and enterotoxin production.

### Effects of Aquatic Products on *Vibrio parahaemolyticus* in Simulated Gastric Fluids

**Figure 3:**
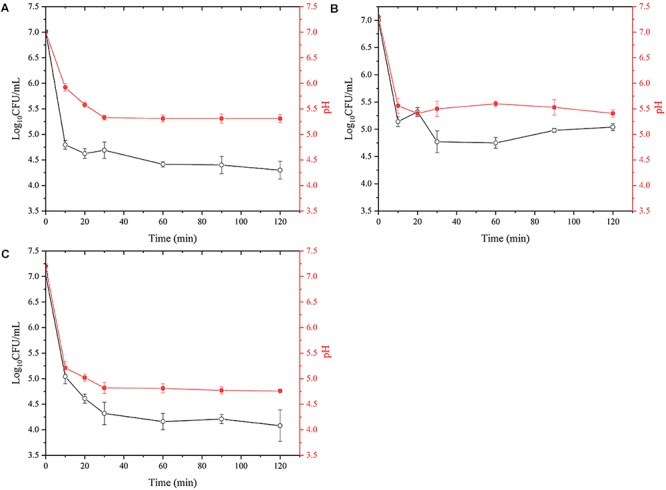
The effect of simulated gastric fluids treatment on the decrease of viable *V. parahaemolyticus* (VPD58) cells in salmon **(A)**, oyster **(B)**, and shrimp **(C)** and the change of pH.

The gastric fluid is maintained at pH 1.6 during fasting and the effect of aquatic products on gastric pH is shown in Figure 3. When the seafood was added to the gastric fluid with a pH of 1.6, the pH of the salmon (A) decreased from the initial value of 7.30 to 5.31, the pH of the oyster (B) decreased from 7.20 to 5.41, and the pH of the shrimp (C) decreased from 7.00 to 4.76. *VPD58* inoculated in aquatic products showed a significant decrease in the number of viable cells in the initial 10 min, and this change in viable numbers correlates well with the change of pH value. After 2 h of treatment, viable cell of *VPD58* inoculated in salmons, oysters, and shrimps were 4.30 Log_10_CFU/mL, 5.04 Log_10_CFU/mL, and 4.08 Log_10_CFU/mL, respectively. However, pure *VPD58* bacterial suspension treated with SGF for 2 h could not be cultured (Supplementary Table 3). This result confirms that seafood matrices can significantly raise the pH level of gastric fluid and assist in *V. parahaemolyticus* surviving passage from the gastric fluid.

### Fate of *Vibrio parahaemolyticus* in an *in vitro* Continuous Digestion Model

The above batch experiments in SSF, SGF and SIF showed that SGF had the highest bactericidal effect on *V. parahaemolyticus*. However, human digestion is a continuous process and we processed the seven strains through an *in vitro* continuous digestion model to validate the changes to survival, morphology and virulence gene expressions (Figure 1A).

**Figure 4:**
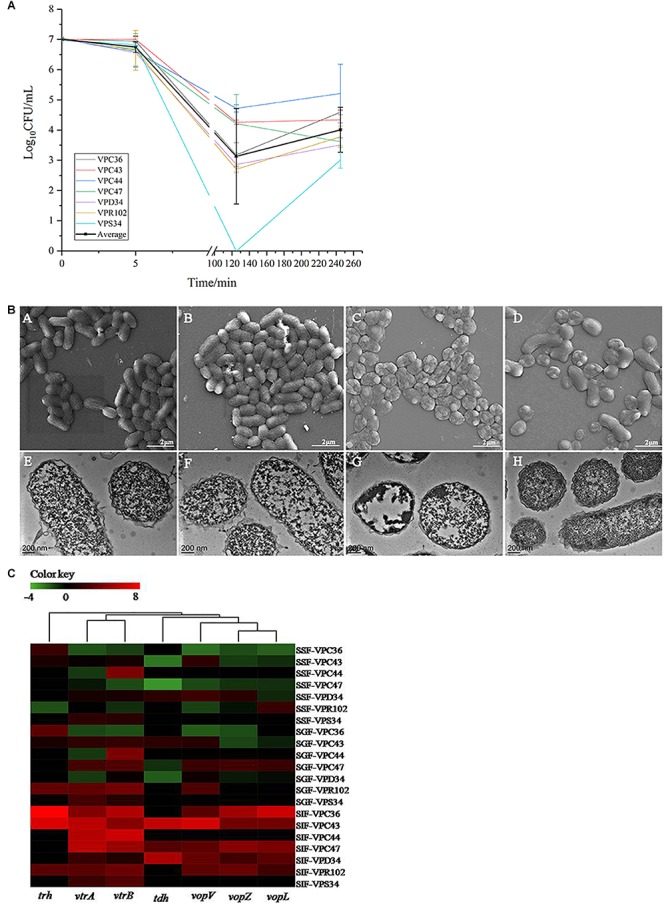
**(A)** The viable cell counts (average ± standard deviation) of *V. parahaemolyticus* during the *in vitro* static digestion model over times, black line represented for average amount. **(B)** Representative photomicrographs of *V. parahaemolyticus in vitro* static digestion model. Untreated group: SEM **(A)** and TEM **(E)**, Saliva phase group: SEM **(B)** and TEM **(F)**, Gastric phase group: SEM **(C)** and TEM **(G)**, Intestinal phase group: SEM **(D)** and TEM **(H)**, respectively. **(C)** Heatmap shows the relative expression of virulence-related genes in *V. parahaemolyticus*. The color codes represent relative expressions, ranging from green (induced expression) to red (repressed expression).

In the continuous model, the viability of *V. parahaemolyticus* did not change significantly after 5 min of passage, where the average cell concentration of all seven strains changed from 7.00 Log_10_CFU/mL to 6.75 Log_10_CFU/mL (Figure 4A). Observations under SEM and TEM also showed no significant difference (Figure 4B). After 125 min of treatment, the survival rate decreased significantly (*p* < 0.01). Average bacterial concentration of seven strains changed from 6.75 Log_10_CFU/mL to 3.13 Log_10_CFU/mL (Figure 4A). The cells were severely shrunk and the cellular contents were released (Figure 4B).

Interestingly, when *V. parahaemolyticus* were treated for 245 min, bacterial concentration of most isolates increased significantly (*P* > 0.05). Average bacterial concentration changed from 3.13 Log_10_CFU/mL to 4.01 Log_10_CFU/mL. The SEM images showed that some of the shrunk cells recovered to a smooth rod shape (Figure 4B). From the TEM pictures (Figure 4B), *V. parahaemolyticus* cells were distributed evenly and the cytoplasm were densely stained, which was different from than those treated with SIF in a batch system.

At the saliva phase, the overall gene expression was down-regulated where only gene *vtrB* in VPC47 was up-regulated 3.58 times. Interestingly, at the gastric phase, gene expression had both positive and negative fold changes, which was significantly different from the all negative fold changes shown in Figure 2I. Additionally at the gastric phase, genes were significantly up-regulated. Information of clustering using the UNIFRAC platform is shown in Figure 4C. The clustered genes are divided into two main branches, where the first branch consists of *trh*, *vtrA* and *vtrB*, and the second branch consists of *tdh, vopV, vopZ*, and *vopL*.

## Discussion

Aquatic products are the main vectors for *V. parahaemolyticus* (Su and Liu, 2007) and the possible mechanism of this pathogenesis was validated in a simulated continuous digestive fluids model system which charts the dynamic changes to *V. parahaemolyticus* in a seafood matrix.

The most efficient barrier to survival and pathogenesis of *V. parahaemolyticus* was the human stomach which plays an important role in killing or inactivating foodborne pathogens. However, food entering the stomach can cause a temporary increase in the stomach pH, ranging from 1.3 to 6.0 (Jennifer et al., 1990). Additionally, Waterman and Small (1998) showed that high protein food can protect bacteria from gastric acid inactivation better than starchy foods. We confirmed this observation for acid sensitive *V. parahaemolyticus* in a seafood matrix.

**Figure 5:**
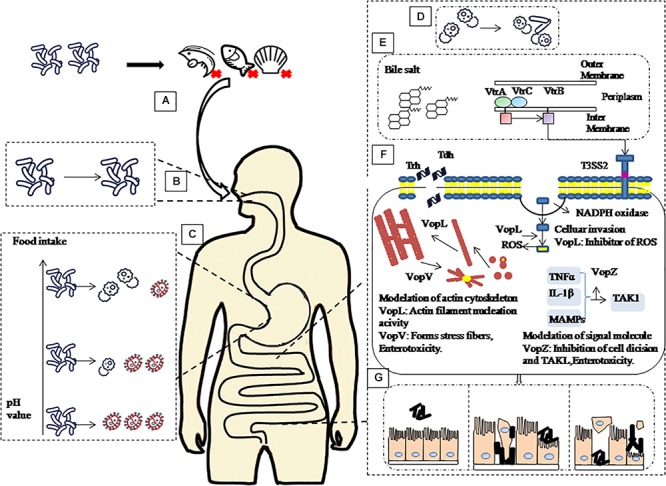
Schematic of pathogenic mechanism of *V. parahaemolyticus* through human digestive tract. **(A)** Contaminated aquatic products are the main source of *V. parahaemolyticus* infection. **(B)** After ingestion, *V. parahaemolyticus* passes through the mouth, but saliva has no significant inactivation effect on it. **(C)** then faces acidic conditions of the stomach before colonizing the small intestine. However, with the increase of gastric fluid pH caused by food intake, the survival rate of *V. parahaemolyticus* in the stomach will increase. **(D)** When *V. parahaemolyticus* reached the intestinal tract, the survival rate increased, and the expression of virulence-related genes increased significantly. **(E)** Under the stimulation of bile salt, VtrA/VtrC complex induced the expression of VtrB, in turn, VtrB activated T3SS2. **(F)** Tdh, Trh and effectors VopL, VopV, VopZ secreted by T3SS2 system infect host cells. **(G)** Ultimately, cause damage to intestinal epithelial cells leads to diseases such as gastroenteritis.

The pH value of the intestine is around 7.0 (Michael, 2013) after gastric emptying and coupled to the body temperature of 37°C, provides an optimal growth environment for *V. parahaemolyticus* (Tang et al., 2015). In this study *V. parahaemolyticus* escaping the stomach environment was resuscitated and expression of the virulence genes was upregulated in the intestinal fluids. Intestinal fluids containing bile salts are known to bind to the VtrA/VtrC complex of *V. parahaemolyticus* and induce VtrB to activate T3SS2 (Li et al., 2016) and *vtrA* and *vtrB* deletion mutants cannot produce TDH and T3SS2-related proteins (Gotoh et al., 2010). VopV, VopZ, and VopL are the three main secretion effectors protein of T3SS2, which play important roles for *V. parahaemolyticus* pathogenicity and colonization in the intestine. Combining all the above results with findings of relevant scholars, we summarize our observation in Figure 5 (Raimondi et al., 2000; Gotoh et al., 2010; Ohnishi et al., 2011; Ham and Orth, 2012; Ritchie et al., 2012; Zhang and Orth, 2013; Li et al., 2016).

In this study, a significant difference was observed in survival rates of pathogenic (*trh+trh+,trh+tdh-,tdh-trh+*) and non-pathogenic (*trh-tdh-*) isolates after SGF treatment. The high survival rate of pathogenic strains in SGF indicates a positive correlation between acid resistance and pathogenicity and highlights heterogeneity in extreme environments. This heterogenous behavior of bacteria in stressful environment was studied by Brian (1998) and includes differences in virulence patterns, growth behavior, inactivation kinetics and biofilm formation capabilities. Delignette-Muller indicated that the heterogeneity of microbial strains can influence conclusions of microbial risk assessments (Delignette-muller and Rosso, 2000).

## Conclusion

In conclusion, this study mainly focused on the survival, morphological changes and virulence gene expression of *V. parahaemolyticus* in simulated digestive fluids. An *in vitro* continuous digestion model was used to validate that *V. parahaemolyticus* contaminating aquatic products is capable of surviving passage through the oral and stomach environment to reach the intestinal tract. With the increase of gastric pH caused by food intake, *V. parahaemolyticus* could escape the bactericidal effect of gastric fluid, resuscitate in the intestine, and express virulence-related genes. This study indicated that seafood matrices can significantly raise the pH level of gastric fluid and thus aids the survival of *V. parahaemolyticus* through passage from human gastric acid and progression of pathogenesis in the intestinal fluid.

## Data Availability

The raw data supporting the conclusions of this manuscript will be made available by the authors, without undue reservation, to any qualified researcher.

## Author Contributions

YZ, YP, and PM conceived and supervised the study. SW, ZZ, and YP conducted the experiments. SW and ZZ analyzed the data and wrote the manuscript. ZZ, PM, YP, and YZ revised the manuscript.

## Conflict of Interest Statement

The authors declare that the research was conducted in the absence of any commercial or financial relationships that could be construed as a potential conflict of interest.
