# Supplementary material for: The Fate of Bacteria in Human Digestive Fluids: A New Perspective Into the Pathogenesis of Vibrio parahaemolyticus
Source: Front Microbiol. 2019 Jul 16;10:1614. doi: 10.3389/fmicb.2019.01614 (PMC6648005; doi:10.3389/fmicb.2019.01614)
Supplement: Supplementary file 1 [file Table_1.DOCX]

***Supplementary Material***

**Supplementary Table 1 The number, genotype, and origin information of sixty strains used in this study**

| No. | | Isolate | | *tdh*^1^ | | *trh*^1^ | | Origin | | No. | | Isolate | | *tdh*^1^ | | *trh*^1^ | | Origin | |
| --- | --- | --- | --- | --- | --- | --- | --- | --- | --- | --- | --- | --- | --- | --- | --- | --- | --- | --- | --- |
| 1 | ATCC17802 | | - | | + | | ATCC | | 31 | | VPD8 | | + | | - | | *Macrobrachium nipponense* | |  |
| 2 | ATCC33847 | | + | | - | | ATCC | | 32 | | VPD14 | | + | | - | | *Macrobrachium nipponense* | |  |
| 3 | VPC1 | | + | | - | | Hospital patient | | 33 | | VPD18 | | + | | - | | *Macrobrachium nipponense* | |  |
| 4 | VPC2 | | + | | - | | Hospital patient | | 34 | | VPD31 | | + | | - | | *Macrobrachium nipponense* | |  |
| 5 | VPC15 | | + | | - | | Hospital patient | | 35 | | VPD33 | | + | | - | | *Penaeus monodon* | |  |
| 6 | VPC16 | | + | | - | | Hospital patient | | 36 | | VPD34 | | + | | - | | *Crassostrea gigas* | |  |
| 7 | VPC17 | | + | | - | | Hospital patient | | 37 | | VPD57 | | + | | - | | *Macrobrachium nipponense* | |  |
| 8 | VPC18 | | - | | + | | Hospital patient | | 38 | | VPD58 | | + | | - | | *Penaeus vannamei* | |  |
| 9 | VPC19 | | + | | - | | Hospital patient | | 39 | | VPD61 | | + | | - | | *Penaeus vannamei* | |  |
| 10 | VPC20 | | + | | - | | Hospital patient | | 40 | | VPR102 | | - | | + | | *Penaeus vannamei* | |  |
| 11 | VPC21 | | + | | - | | Hospital patient | | 41 | | VPR103 | | - | | + | | *Penaeus vannamei* | |  |
| 12 | VPC22 | | + | | - | | Hospital patient | | 42 | | VPR104 | | - | | + | | *Penaeus vannamei* | |  |
| 13 | VPC26 | | + | | - | | Hospital patient | | 43 | | VPR105 | | - | | + | | *Penaeus vannamei* | |  |
| 14 | VPC27 | | + | | - | | Hospital patient | | 44 | | VPR106 | | - | | + | | *Penaeus vannamei* | |  |
| 15 | VPC28 | | + | | - | | Hospital patient | | 45 | | VPR107 | | - | | + | | *Penaeus vannamei* | |  |
| 16 | VPC29 | | + | | - | | Hospital patient | | 46 | | VPR108 | | - | | + | | *Penaeus vannamei* | |  |
| 17 | VPC32 | | + | | - | | Hospital patient | | 47 | | VPR110 | | - | | + | | *Penaeus vannamei* | |  |
| 18 | VPC33 | | + | | - | | Hospital patient | | 48 | | VPR111 | | - | | + | | *Penaeus vannamei* | |  |
| 19 | VPC34 | | + | | - | | Hospital patient | | 49 | | VPS16 | | - | | - | | *Macrobrachium nipponense* | |  |
| 20 | VPC35 | | + | | - | | Hospital patient | | 50 | | VPS17 | | - | | - | | *Macrobrachium nipponense* | |  |
| 21 | VPC36 | | - | | + | | Hospital patient | | 51 | | VPS18 | | - | | - | | *Macrobrachium nipponense* | |  |
| 22 | VPC43 | | + | | + | | Hospital patient | | 52 | | VPS19 | | - | | - | | *Macrobrachium nipponense* | |  |
| 23 | VPC44 | | - | | - | | Hospital patient | | 53 | | VPS20 | | - | | - | | *Macrobrachium nipponense* | |  |
| 24 | VPC47 | | + | | - | | Hospital patient | | 54 | | VPS21 | | - | | - | | *Macrobrachium nipponense* | |  |
| 25 | VPC48 | | + | | - | | Hospital patient | | 55 | | VPS33 | | - | | - | | *Macrobrachium nipponense* | |  |
| 26 | VPC49 | | + | | + | | Hospital patient | | 56 | | VPS34 | | - | | - | | *Macrobrachium nipponense* | |  |
| 27 | VPC54 | | + | | + | | Hospital patient | | 57 | | VPS35 | | - | | - | | *Macrobrachium nipponense* | |  |
| 28 | VPC85 | | - | | + | | Hospital patient | | 58 | | VPS36 | | - | | - | | *Macrobrachium nipponense* | |  |
| 29 | VPC89 | | + | | - | | Hospital patient | | 59 | | VPS37 | | - | | - | | *Macrobrachium nipponense* | |  |
| 30 | VPC94 | | - | | + | | Hospital patient | | 60 | | VPS38 | | - | | - | | *Macrobrachium nipponense* | |  |

Note: *tdh* and *trh* are genes that incode TDH and TRH. “+” represents positive genotypic, and “−” means negative genotypic.
