# Supplementary material for: The Fate of Bacteria in Human Digestive Fluids: A New Perspective Into the Pathogenesis of Vibrio parahaemolyticus
Source: Front Microbiol. 2019 Jul 16;10:1614. doi: 10.3389/fmicb.2019.01614 (PMC6648005; doi:10.3389/fmicb.2019.01614)
Supplement: Supplementary file 2 [file Table_2.DOCX]

***Supplementary Material***

**Supplementary Table 2 Survival rate of *V. parahaemolyticus* in simulated saliva fluid**

| NO. | Control  Log_10_CFU/mL | SSF  Log_10_CFU/mL | Survival rate ( %) | NO. | Control  Log_10_CFU/mL | SSF  Log_10_CFU/mL | Survival rate (%) | NO. | Control  Log_10_CFU/mL | SSF  Log_10_CFU/mL | Survival rate (%) |
| --- | --- | --- | --- | --- | --- | --- | --- | --- | --- | --- | --- |
| 1 | 7.00±0.02 | 7.01±0.05 | 100.14±0.47 | 21 | 7.01±0.04 | 6.94±0.15 | 99.00±0.29 | 41 | 7.00±0.01 | 6.66±0.61 | 95.14±0.56 |
| 2 | 7.01±0.01 | 6.95±0.03 | 99.14±1.11 | 22 | 6.99±0.01 | 7.00±0.11 | 100.14±0.34 | 42 | 7.01±0.02 | 7.43±0.07 | 105.99±0.28 |
| 3 | 7.02±0.04 | 6.55±0.08 | 93.30±0.59 | 23 | 7.00±0.02 | 6.60±0.52 | 94.29±0.16 | 43 | 7.01±0.04 | 6.89±0.28 | 98.29±0.39 |
| 4 | 7.00±0.02 | 6.34±0.20 | 90.57±0.23 | 24 | 7.01±0.06 | 6.66±0.53 | 95.01±0.81 | 44 | 7.02±0.01 | 7.34±0.06 | 104.56±0.24 |
| 5 | 7.01±0.03 | 6.58±0.04 | 93.87±0.14 | 25 | 7.01±0.02 | 6.88±0.03 | 98.15±0.83 | 45 | 7.03±0.04 | 6.78±0.11 | 96.44±0.51 |
| 6 | 7.01±0.05 | 6.87±0.11 | 98.00±0.36 | 26 | 7.03±0.02 | 6.43±0.19 | 91.47±0.67 | 46 | 7.01±0.02 | 7.32±0.36 | 104.42±0.53 |
| 7 | 7.01±0.04 | 6.91±0.12 | 98.57±0.51 | 27 | 7.00±0.05 | 6.92±0.01 | 98.86±0.59 | 47 | 7.03±0.01 | 7.41±0.16 | 105.41±0.43 |
| 8 | 7.03±0.01 | 6.83±0.03 | 97.16±0.24 | 28 | 6.98±0.01 | 6.61±0.28 | 94.70±1.10 | 48 | 7.02±0.03 | 6.28±0.09 | 89.46±0.44 |
| 9 | 7.05±0.01 | 7.28±0.07 | 103.26±0.16 | 29 | 7.01±0.03 | 7.23±0.14 | 103.14±0.49 | 49 | 7.01±0.04 | 6.31±0.17 | 90.01±0.61 |
| 10 | 6.99±0.06 | 7.11±0.11 | 101.72±0.31 | 30 | 7.03±0.02 | 6.71±0.59 | 95.45±1.21 | 50 | 7.02±0.04 | 6.56±0.09 | 93.45±0.51 |
| 11 | 7.01±0.02 | 6.90±0.15 | 98.43±0.27 | 31 | 7.00±0.03 | 6.69±0.01 | 95.57±0.56 | 51 | 7.00±0.06 | 6.88±0.01 | 98.29±0.29 |
| 12 | 7.01±0.01 | 6.89±0.11 | 98.29±0.39 | 32 | 7.00±0.04 | 6.62±0.37 | 94.57±0.76 | 52 | 6.99±0.02 | 6.56±0.21 | 93.85±1.13 |
| 13 | 7.00±0.02 | 6.58±0.08 | 94.00±0.51 | 33 | 7.01±0.04 | 6.83±0.06 | 97.43±0.51 | 53 | 7.00±0.05 | 6.83±0.11 | 97.57±0.24 |
| 14 | 7.01±0.05 | 7.18±0.31 | 102.43±0.61 | 34 | 6.99±0.05 | 6.83±0.08 | 97.71±0.60 | 54 | 7.00±0.01 | 6.78±0.70 | 96.86±0.78 |
| 15 | 7.00±0.01 | 6.83±0.23 | 97.57±1.24 | 35 | 7.01±0.01 | 6.56±0.46 | 93.58±0.46 | 55 | 7.04±0.05 | 6.91±0.10 | 98.15±0.27 |
| 16 | 6.98±0.01 | 6.84±0.07 | 97.99±1.31 | 36 | 7.01±0.06 | 6.31±0.17 | 90.01±0.57 | 56 | 6.97±0.06 | 6.83±0.05 | 97.99±0.61 |
| 17 | 7.01±0.03 | 6.59±0.28 | 94.01±0.16 | 37 | 7.02±0.01 | 6.67±0.24 | 95.01±0.34 | 57 | 7.02±0.01 | 6.74±0.16 | 96.01±0.59 |
| 18 | 7.01±0.04 | 6.84±0.18 | 97.57±0.59 | 38 | 7.09±0.02 | 6.60±0.57 | 93.09±0.16 | 58 | 6.98±0.03 | 6.86±0.19 | 98.28±0.24 |
| 19 | 7.03±0.05 | 6.86±0.03 | 97.58±2.01 | 39 | 7.01±0.04 | 6.58±0.58 | 93.87±1.24 | 59 | 7.01±0.05 | 6.69±0.27 | 95.44±0.36 |
| 20 | 7.00±0.09 | 6.90±0.17 | 98.57±0.16 | 40 | 7.04±0.05 | 6.64±0.66 | 94.32±1.16 | 60 | 7.01±0.04 | 6.49±0.38 | 92.58±0.27 |
