# Supplementary material for: The Fate of Bacteria in Human Digestive Fluids: A New Perspective Into the Pathogenesis of Vibrio parahaemolyticus
Source: Front Microbiol. 2019 Jul 16;10:1614. doi: 10.3389/fmicb.2019.01614 (PMC6648005; doi:10.3389/fmicb.2019.01614)
Supplement: Supplementary file 3 [file Table_3.DOCX]

***Supplementary Material***

**Supplementary Table 3 Survival rate of *V. parahaemolyticus* in simulated gastric fluid**

| NO. | Control | SGF | | | | | |
| --- | --- | --- | --- | --- | --- | --- | --- |
|  | Log_10_CFU/mL | pH=2.0 | Surivival rate (%) | pH=3.0 | Surivival rate (%) | pH=4.0 | Surivival rate (%) |
| 1 | 7.00±0.02 | 0^1^ | 0.00±0.00 | 0^1^ | 0.00±0.00 | 2.48±0.04 | 35.43±1.34 |
| 2 | 7.01±0.01 | 0^1^ | 0.00±0.00 | 0^1^ | 0.00±0.00 | 2.69±0.15 | 38.37±1.21 |
| 3 | 7.02±0.04 | 0^1^ | 0.00±0.00 | 0^1^ | 0.00±0.00 | 2.50±0.01 | 35.61±1.37 |
| 4 | 7.00±0.02 | 0^1^ | 0.00±0.00 | 0^1^ | 0.00±0.00 | 2.91±0.47 | 41.57±1.28 |
| 5 | 7.01±0.03 | 0^1^ | 0.00±0.00 | 0^1^ | 0.00±0.00 | 2.90±0.19 | 41.37±0.92 |
| 6 | 7.01±0.05 | 0^1^ | 0.00±0.00 | 0^1^ | 0.00±0.00 | 2.97±0.24 | 42.37±0.43 |
| 7 | 7.01±0.04 | 0^1^ | 0.00±0.00 | 0^1^ | 0.00±0.00 | 0^1^ | 0.00±0.00 |
| 8 | 7.03±0.01 | 0^1^ | 0.00±0.00 | 0^1^ | 0.00±0.00 | 3.15±0.63 | 44.81±0.61 |
| 9 | 7.05±0.01 | 0^1^ | 0.00±0.00 | 0^1^ | 0.00±0.00 | 3.28±0.09 | 46.52±0.72 |
| 10 | 6.99±0.06 | 0^1^ | 0.00±0.00 | 0^1^ | 0.00±0.00 | 3.09±0.16 | 44.21±1.61 |
| 11 | 7.01±0.02 | 0^1^ | 0.00±0.00 | 0^1^ | 0.00±0.00 | 0^1^ | 0.00±0.00 |
| 12 | 7.01±0.01 | 0^1^ | 0.00±0.00 | 0^1^ | 0.00±0.00 | 2.74±0.22 | 39.09±0.86 |
| 13 | 7.00±0.02 | 0^1^ | 0.00±0.00 | 0^1^ | 0.00±0.00 | 0^1^ | 0.00±0.00 |
| 14 | 7.01±0.05 | 0^1^ | 0.00±0.00 | 0^1^ | 0.00±0.00 | 2.62±0.76 | 37.38±0.56 |
| 15 | 7.00±0.01 | 0^1^ | 0.00±0.00 | 0^1^ | 0.00±0.00 | 3.15±0.24 | 45.00±0.81 |
| 16 | 6.98±0.01 | 0^1^ | 0.00±0.00 | 0^1^ | 0.00±0.00 | 0^1^ | 0.00±0.00 |
| 17 | 7.01±0.03 | 0^1^ | 0.00±0.00 | 0^1^ | 0.00±0.00 | 2.53±0.15 | 36.09±0.73 |
| 18 | 7.01±0.04 | 0^1^ | 0.00±0.00 | 0^1^ | 0.00±0.00 | 2.74±0.45 | 39.09±1.15 |
| 19 | 7.03±0.05 | 0^1^ | 0.00±0.00 | 0^1^ | 0.00±0.00 | 0^1^ | 0.00±0.00 |
| 20 | 7.00±0.09 | 0^1^ | 0.00±0.00 | 0^1^ | 0.00±0.00 | 0^1^ | 14.29±1.27 |
| 21 | 7.01±0.04 | 0^1^ | 0.00±0.00 | 0^1^ | 0.00±0.00 | 2.81±0.40 | 40.09±0.43 |
| 22 | 6.99±0.01 | 0^1^ | 0.00±0.00 | 0^1^ | 0.00±0.00 | 3.21±0.07 | 45.92±1.24 |
| 23 | 7.00±0.02 | 0^1^ | 0.00±0.00 | 0^1^ | 0.00±0.00 | 3.62±0.12 | 51.71±1.03 |
| 24 | 7.01±0.06 | 0^1^ | 0.00±0.00 | 0^1^ | 0.00±0.00 | 3.49±0.67 | 49.79±0.82 |
| 25 | 7.01±0.02 | 0^1^ | 0.00±0.00 | 0^1^ | 0.00±0.00 | 0^1^ | 0.00±0.00 |
| 26 | 7.03±0.02 | 0^1^ | 0.00±0.00 | 0^1^ | 0.00±0.00 | 3.75±1.30 | 53.34±1.16 |
| 27 | 7.00±0.05 | 0^1^ | 0.00±0.00 | 0^1^ | 0.00±0.00 | 3.68±1.29 | 52.57±0.81 |
| 28 | 6.98±0.01 | 0^1^ | 0.00±0.00 | 0^1^ | 0.00±0.00 | 3.12±0.11 | 44.70±0.83 |
| 29 | 7.01±0.03 | 0^1^ | 0.00±0.00 | 0^1^ | 0.00±0.00 | 0^1^ | 0.00±0.00 |
| 30 | 7.03±0.02 | 0^1^ | 0.00±0.00 | 0^1^ | 0.00±0.00 | 3.44±0.38 | 48.93±1.10 |
| 31 | 7.00±0.03 | 0^1^ | 0.00±0.00 | 0^1^ | 0.00±0.00 | 2.72±0.07 | 38.86±1.16 |
| 32 | 7.00±0.04 | 0^1^ | 0.00±0.00 | 0^1^ | 0.00±0.00 | 0^1^ | 0.00±0.00 |
| 33 | 7.01±0.04 | 0^1^ | 0.00±0.00 | 0^1^ | 0.00±0.00 | 2.70±0.52 | 38.52±1.92 |
| 34 | 6.99±0.05 | 0^1^ | 0.00±0.00 | 0^1^ | 0.00±0.00 | 0^1^ | 0.00±0.00 |
| 35 | 7.01±0.01 | 0^1^ | 0.00±0.00 | 0^1^ | 0.00±0.00 | 2.60±0.24 | 37.09±1.90 |
| 36 | 7.01±0.06 | 0^1^ | 0.00±0.00 | 0^1^ | 0.00±0.00 | 3.11±0.29 | 44.37±1.13 |
| 37 | 7.02±0.01 | 0^1^ | 0.00±0.00 | 0^1^ | 0.00±0.00 | 0^1^ | 0.00±0.00 |
| 38 | 7.09±0.02 | 0^1^ | 0.00±0.00 | 0^1^ | 0.00±0.00 | 0^1^ | 0.00±0.00 |
| 39 | 7.01±0.04 | 0^1^ | 0.00±0.00 | 0^1^ | 0.00±0.00 | 0^1^ | 0.00±0.00 |
| 40 | 7.04±0.05 | 0^1^ | 0.00±0.00 | 0^1^ | 0.00±0.00 | 2.12±0.10 | 30.11±1.11 |
| 41 | 7.00±0.01 | 0^1^ | 0.00±0.00 | 0^1^ | 0.00±0.00 | 0^1^ | 0.00±0.00 |
| 42 | 7.01±0.02 | 0^1^ | 0.00±0.00 | 0^1^ | 0.00±0.00 | 3.62±0.49 | 51.64±0.78 |
| 43 | 7.01±0.04 | 0^1^ | 0.00±0.00 | 0^1^ | 0.00±0.00 | 3.38±0.12 | 48.22±0.93 |
| 44 | 7.02±0.01 | 0^1^ | 0.00±0.00 | 0^1^ | 0.00±0.00 | 3.84±0.96 | 54.70±1.11 |
| 45 | 7.03±0.04 | 0^1^ | 0.00±0.00 | 0^1^ | 0.00±0.00 | 3.11±0.05 | 44.24±1.17 |
| 46 | 7.01±0.02 | 0^1^ | 0.00±0.00 | 0^1^ | 0.00±0.00 | 3.49±0.64 | 49.79±0.89 |
| 47 | 7.03±0.01 | 0^1^ | 0.00±0.00 | 0^1^ | 0.00±0.00 | 3.11±0.53 | 44.24±0.69 |
| 48 | 7.02±0.03 | 0^1^ | 0.00±0.00 | 0^1^ | 0.00±0.00 | 0^1^ | 0.00±0.00 |
| 49 | 7.01±0.04 | 0^1^ | 0.00±0.00 | 0^1^ | 0.00±0.00 | 0^1^ | 0.00±0.00 |
| 50 | 7.02±0.04 | 0^1^ | 0.00±0.00 | 0^1^ | 0.00±0.00 | 0^1^ | 0.00±0.00 |
| 51 | 7.00±0.06 | 0^1^ | 0.00±0.00 | 0^1^ | 0.00±0.00 | 0^1^ | 0.00±0.00 |
| 52 | 6.99±0.02 | 0^1^ | 0.00±0.00 | 0^1^ | 0.00±0.00 | 0^1^ | 0.00±0.00 |
| 53 | 7.00±0.05 | 0^1^ | 0.00±0.00 | 0^1^ | 0.00±0.00 | 0^1^ | 0.00±0.00 |
| 54 | 7.00±0.01 | 0^1^ | 0.00±0.00 | 0^1^ | 0.00±0.00 | 0^1^ | 0.00±0.00 |
| 55 | 7.04±0.05 | 0^1^ | 0.00±0.00 | 0^1^ | 0.00±0.00 | 0^1^ | 0.00±0.00 |
| 56 | 6.97±0.06 | 0^1^ | 0.00±0.00 | 0^1^ | 0.00±0.00 | 0^1^ | 0.00±0.00 |
| 57 | 7.02±0.01 | 0^1^ | 0.00±0.00 | 0^1^ | 0.00±0.00 | 0^1^ | 0.00±0.00 |
| 58 | 6.98±0.03 | 0^1^ | 0.00±0.00 | 0^1^ | 0.00±0.00 | 0^1^ | 0.00±0.00 |
| 59 | 7.01±0.05 | 0^1^ | 0.00±0.00 | 0^1^ | 0.00±0.00 | 0^1^ | 0.00±0.00 |
| 60 | 7.01±0.04 | 0^1^ | 0.00±0.00 | 0^1^ | 0.00±0.00 | 0^1^ | 0.00±0.00 |

1:NC: non-culturable
