# Supplementary material for: The Fate of Bacteria in Human Digestive Fluids: A New Perspective Into the Pathogenesis of Vibrio parahaemolyticus
Source: Front Microbiol. 2019 Jul 16;10:1614. doi: 10.3389/fmicb.2019.01614 (PMC6648005; doi:10.3389/fmicb.2019.01614)
Supplement: Supplementary file 4 [file Table_4.DOCX]

***Supplementary Material***

**Supplementary Table 4 Survival rate of *V. parahaemolyticus* in simulated intestinal fluid**

| NO. | Control  Log_10_CFU/mL | SIF  Log_10_CFU/mL | Survival rate ( %) | NO. | Control  Log_10_CFU/mL | SIF  Log_10_CFU/mL | Survival rate(%) | NO. | Control  Log_10_CFU/mL | SIF  Log_10_CFU/mL | Survival rate (%) |
| --- | --- | --- | --- | --- | --- | --- | --- | --- | --- | --- | --- |
| 1 | 7.00±0.02 | 6.15±0.13 | 87.86±0.35 | 21 | 7.01±0.04 | 6.56±0.04 | 93.58±0.34 | 41 | 7.00±0.01 | 4.78±0.61 | 68.29±0.61 |
| 2 | 7.01±0.01 | 5.91±0.24 | 84.31±0.41 | 22 | 6.99±0.01 | 6.08±0.16 | 86.98±0.24 | 42 | 7.01±0.02 | 5.59±0.06 | 79.74±0.51 |
| 3 | 7.02±0.04 | 5.13±0.09 | 73.08±0.61 | 23 | 7.00±0.02 | 6.63±0.27 | 94.71±0.48 | 43 | 7.01±0.04 | 5.37±0.19 | 76.60±0.52 |
| 4 | 7.00±0.02 | 5.63±0.31 | 80.43±0.20 | 24 | 7.01±0.06 | 5.53±0.09 | 78.89±0.36 | 44 | 7.02±0.01 | 4.88±0.21 | 69.52±0.41 |
| 5 | 7.01±0.03 | 4.40±0.29 | 62.77±0.31 | 25 | 7.01±0.02 | 5.27±0.23 | 75.18±0.51 | 45 | 7.03±0.04 | 4.59±0.41 | 65.29±0.61 |
| 6 | 7.01±0.05 | 5.15±0.33 | 73.47±0.38 | 26 | 7.03±0.02 | 6.76±0.16 | 96.16±0.19 | 46 | 7.01±0.02 | 5.98±0.17 | 85.31±0.22 |
| 7 | 7.01±0.04 | 5.48±0.49 | 78.17±0.52 | 27 | 7.00±0.05 | 6.31±0.42 | 90.14±0.37 | 47 | 7.03±0.01 | 4.89±0.03 | 69.56±0.49 |
| 8 | 7.03±0.01 | 5.78±0.36 | 82.22±0.16 | 28 | 6.98±0.01 | 6.15±0.34 | 88.11±0.28 | 48 | 7.02±0.03 | 6.34±0.06 | 90.31±0.35 |
| 9 | 7.05±0.01 | 5.32±0.29 | 75.46±0.34 | 29 | 7.01±0.03 | 6.04±0.29 | 86.16±0.51 | 49 | 7.01±0.04 | 5.41±0.22 | 77.18±0.28 |
| 10 | 6.99±0.06 | 5.93±0.66 | 84.84±0.22 | 30 | 7.03±0.02 | 6.45±0.11 | 91.75±0.51 | 50 | 7.02±0.04 | 6.66±0.15 | 94.87±0.62 |
| 11 | 7.01±0.02 | 5.23±0.31 | 74.61±0.50 | 31 | 7.00±0.03 | 5.08±0.31 | 72.57±0.38 | 51 | 7.00±0.06 | 5.56±0.23 | 79.43±0.66 |
| 12 | 7.01±0.01 | 6.03±0.08 | 86.02±0.61 | 32 | 7.00±0.04 | 5.52±0.22 | 78.86±0.42 | 52 | 6.99±0.02 | 5.20±0.18 | 74.39±0.57 |
| 13 | 7.00±0.02 | 6.79±0.14 | 97.00±0.34 | 33 | 7.01±0.04 | 5.18±0.06 | 73.89±0.51 | 53 | 7.00±0.05 | 5.28±0.08 | 75.43±0.46 |
| 14 | 7.01±0.05 | 6.14±0.41 | 87.59±0.21 | 34 | 6.99±0.05 | 4.72±0.36 | 67.53±0.88 | 54 | 7.00±0.01 | 4.79±0.17 | 68.43±0.39 |
| 15 | 7.00±0.01 | 5.94±0.19 | 84.86±0.61 | 35 | 7.01±0.01 | 5.36±0.27 | 76.46±1.02 | 55 | 7.04±0.05 | 5.12±0.13 | 72.73±1.04 |
| 16 | 6.98±0.01 | 4.97±0.38 | 71.20±0.25 | 36 | 7.01±0.06 | 3.98±0.44 | 56.78±0.81 | 56 | 6.97±0.06 | 4.87±0.05 | 69.87±1.11 |
| 17 | 7.01±0.03 | 5.22±0.15 | 74.47±0.33 | 37 | 7.02±0.01 | 4.17±0.09 | 59.40±0.61 | 57 | 7.02±0.01 | 5.10±0.02 | 72.65±0.61 |
| 18 | 7.01±0.04 | 4.76±0.08 | 67.90±0.64 | 38 | 7.09±0.02 | 4.29±0.32 | 60.51±0.24 | 58 | 6.98±0.03 | 5.71±0.19 | 81.81±0.67 |
| 19 | 7.03±0.05 | 5.92±0.21 | 84.21±0.68 | 39 | 7.01±0.04 | 4.59±0.15 | 65.48±0.51 | 59 | 7.01±0.05 | 5.22±0.61 | 74.47±0.53 |
| 20 | 7.00±0.09 | 5.32±0.11 | 76.00±0.49 | 40 | 7.04±0.05 | 4.48±0.72 | 63.64±0.33 | 60 | 7.01±0.04 | 4.63±0.28 | 66.05±0.46 |
